# Elder abuse: a conceptual analysis

**DOI:** 10.1590/0034-7167-2023-0150

**Published:** 2023-12-04

**Authors:** Renata Clemente dos Santos-Rodrigues, Gleicy Karine Nascimento de Araújo-Monteiro, Ana Márcia Nóbrega Dantas, Patrícia Josefa Fernandes Beserra, Ronei Marcos de Morais, Rafaella Queiroga Souto

**Affiliations:** I Universidade Estadual da Paraíba. Campina Grande, Paraíba, Brazil; II Universidade Federal de Alagoas. Maceió, Alagoas, Brazil.; III Universidade Federal da Paraíba. João Pessoa, Paraíba, Brazil

**Keywords:** Aged, Violence, Elder Abuse, Exposure to Violence, Concept Formation, Anciano, Violencia, Abuso de Ancianos, Exposición a la Violencia, Formación de Concepto, Idoso, Violência, Abuso de Idosos, Exposição à, Violência, Formação de Conceito

## Abstract

**Objective:**

to analyze the concept of elder abuse and identify its respective antecedents, attributes and consequents.

**Methods:**

this is a conceptual analysis according to Walker and Avant’s proposition. The search for the concept was mediated by an integrative review in the LILACS, PubMed, CINAHL, Web of Science and BDENF databases.

**Results:**

as antecedents, female, family member, low social support and low income or socioeconomic conditions stand out. Attributes were threats and/or intimidation, intentional use of physical force, using resources without authorization, unwanted sexual activity, offering low insufficient amount of nutrients for older adults and not meeting older adults’ affective/emotional needs. Consequents were psychological disorders, dependence on aggressors, environment of insecurity and damage/loss of human rights or human dignity.

**Final considerations:**

the phenomenon under discussion is broad and multifaceted, suggesting expansion of studies related to the theme in order to explore it in detail.

## RESUMO

**Objetivo:**

analisar o conceito de violência contra pessoa idosa e identificar seus respectivos antecedentes, atributos e consequentes.

**Métodos:**

análise de conceito, de acordo com a proposição de Walker e Avant. A busca pelo conceito foi mediada por uma revisão integrativa nas bases LILACS, PubMed, CINAHL, *Web of Science* e BDENF.

**Resultados:**

como antecedentes, destacam-se sexo feminino, membro da família, baixo apoio social e baixa renda ou condições socioeconômicas. Os atributos foram ameaças e/ou intimidações, uso intencional da força física, usar recursos sem sua autorização, atividade sexual indesejada, ofertar baixa quantidade de nutrientes insuficientes ao idoso e não atendimento as necessidades afetivas/emocionais do idoso. Os consequentes foram transtornos psicológicos, dependência do agressor, ambiente de insegurança e danos/perda dos direitos humanos ou a dignidade humana.

**Considerações finais:**

o fenômeno em discussão é amplo e multifacetado, sugerindo ampliação de estudos relacionados à temática a fim de explorá-la minunciosamente.

## RESUMEN

**Objetivo:**

analizar el concepto de violencia contra el anciano e identificar sus respectivos antecedentes, atributos y consecuencias.

**Métodos:**

análisis de concepto, según propuesta de Walker y Avant. La búsqueda del concepto estuvo mediada por una revisión integradora en las bases de datos LILACS, PubMed, CINAHL, Web of Science y BDENF.

**Resultados:**

como antecedentes, se destacan el género femenino, familiar, bajo apoyo social y bajos ingresos o condiciones socioeconómicas. Los atributos fueron amenazas y/o intimidación, uso intencional de la fuerza física, uso de recursos sin su autorización, actividad sexual no deseada, oferta insuficiente de nutrientes al anciano y no satisfacción de las necesidades afectivas/emocionales del anciano. Las consecuencias fueron trastornos psicológicos, dependencia del agresor, ambiente de inseguridad y daño/pérdida de los derechos humanos o de la dignidad humana.

**Consideraciones finales:**

el fenómeno en discusión es amplio y multifacético, sugiriendo la ampliación de estudios relacionados con el tema para explorarlo en detalle.

## INTRODUCTION

Population aging increases rapidly over the years due to the decline in birth and fertility indicators associated with the increase in the general population’s life expectancy. As individuals age, they experience behavioral, physical, psychological, cognitive and social changes of a physiological nature, but that have the potential for the emergence of comorbidities that can result in fragility, loss of autonomy and physical dependence, thus making them exposed to experiencing situations of violence^(1)^.

Although it is a worldwide phenomenon, the synthesis of quantitative data on the occurrence of elder abuse (EA) is rare, especially in lowand middle-income countries. The Lancet Global Health published in a meta-analysis that approximately one in every six older adults suffer some type of violence, distributed with an overall prevalence of 15.7%, in addition to 11.6% related to psychological violence, 6.8%, financial abuse, 4.2%, negligence-type violence, 2.6%, physical violence, and 0.9%, sexual violence^(2)^. Considering Brazil’s regional variability, in the Northeast, physical violence is considered the most prevalent (28.0%), followed by negligence and abandonment (17.3%)^(3)^. However, in a study developed with reports generated by Dial 100, negligence was the most predominant (37%), followed by psychological (27%) and financial (20.3%), and sexual violence had a low number of reports (0.3%)^(4)^.

Its definition is generically defined as any intentional or unintentional act that causes harm and suffering to older adults, resulting in a decline in quality of life, increased risk of physical and emotional illness, beyond the susceptibility to face the most varied types of violence^(5)^; therefore, it is considered a public health problem that affects any older adult, regardless of social class, ethnicity or religion.

By observing the dimension of the phenomenon in EA, it is possible to perceive its multifaceted and multidimensional character, in addition to various social and individual developments caused as a consequence of its occurrence^(6)^. Conceptualizing it then becomes a complex and abstract task, since the definition of EA is not very enlightening considering the amplitude and the relationship of the phenomenon with cultural, religious and regional characteristics^(7)^. It is therefore vital that the concept be better explored in order to understand what are its defining characteristics, antecedents and consequents that affect older adults.

The elaboration of concepts can be considered the basis of knowledge and scientific development for the construction of theoretical models that deal with the definition of fields of action, methods and objects of study clearly^(8)^. They can also represent the abstract reality of cognitive experiences^(9)^.

Carrying out concept analyzes is considered extremely useful to unveil phenomena and can be performed using several methods, however, in nursing, the model proposed by Walker and Avant is widely disseminated^(9)^. This model aims to strengthen the theoretical basis regarding the object of study. It also provides subsidy for the elaboration of psychometric instruments through the list of defining attributes (characteristics that define the object of study), antecedents (events or incidents that happen before the occurrence of the phenomenon) and consequents (are results of the occurrence of concept)^(8)^.

The present study was conceived through the question: what are the essential attributes, antecedents and consequents that clarify the definition of EA according to Walker’s and Avant’s method?

## OBJECTIVE

To analyze the concept of EA and identify its respective antecedents, attributes and consequents.

## METHODS

### Ethical aspects

The study does not need to be assessed by an ethics committee for its execution, considering that it used the literature available in databases, not directly or indirectly involving human beings.

### Theoretical-methodological framework

The theoretical-methodological framework used was the concept analysis model proposed by Walker and Avant. The model has eight interactive steps: concept selection; outlining the objectives of analysis; identification of possible uses of the concept; determination of essential attributes; model case identification; otherwise identification; identification of the antecedents and consequents of the concept; definition of the empirical references of the studied concept^(8)^.

The explanation for occurrence of violence needs to be observed from several facets, as there is no single factor that explains violent actions and relationships. From this point of view, the use of the ecological model proposed by Bronfenbrenner in 1975 for categorization of data related to the antecedents and consequents of EA is justified, because, in addition to providing a better understanding of the phenomenon, it subsidizes the identification of relationships between individual (personal factors perceived in behavior), relational (close social relationships), community (community contexts) and social (wider social factors) dimensions^(10)^.

### Study design

This is a conceptual analysis study, whose purpose is to distinguish, refine ambiguities and clarify concepts. The method makes it possible to analyze the structure and function of the basic elements of the concept of EA.

### Methodological procedures

#### Study setting

The study was carried out by researchers from the Group of Studies and Research in Forensic Nursing (GEPEFO - *Grupo de Estudos e Pesquisa em Enfermagem Forense*), linked to the *Universidade Federal da Paraíba* (UFPB), as an initial step in the construction and validity of a scale for screening EA: thesis product of the main author of this study. Analyzing the concept is essential for instruments to be applicable in care practice, considering that the concept clarification provides greater precision in identifying the phenomenon in older adults.

#### Data collection and organization

In the first stage, the concept of EA was selected, allowing professionals to broaden their understanding of situations of violence or risk of violence, and, in the second stage, the objective described in the Objectives section was defined. In the third stage, a thorough search was carried out to identify the use of the concept in the literature. For this, the six stages of integrative review were carried out^(11)^.

Data came from a research question elaborated according to the PCC mnemonic, in which P (patient) - older adult person, C (concept) - concept of EA and C (context) - attributes, antecedents and consequents of EA. What are the concepts presented in the literature to define EA? What are the attributes, antecedents and consequents of the phenomenon?

Studies published between 2012 and 2022, developed with a theme related to people aged 60 years or older, written in Portuguese, English and Spanish, available in full electronically, were included. Documents classified as gray literature (editorials, newsletters, news, theses and dissertations) and duplicates were excluded.

The search was carried out in the Latin American and Caribbean databases in Health Sciences (LILACS), BDENF, MEDLINE via PubMed, Cumulative Index to Nursing and Allied Health Literature (CINAHL) and Web of Science, using the descriptors: “Aged”, “Exposure to violence”, “Elder Abuse”. The selected descriptors were combined using Boolean operators, in order to enable the retrieval of documents, and then the general combination was adopted: “Aged” AND “Exposure to violence” OR “Elder Abuse”.

**Figure 1:**
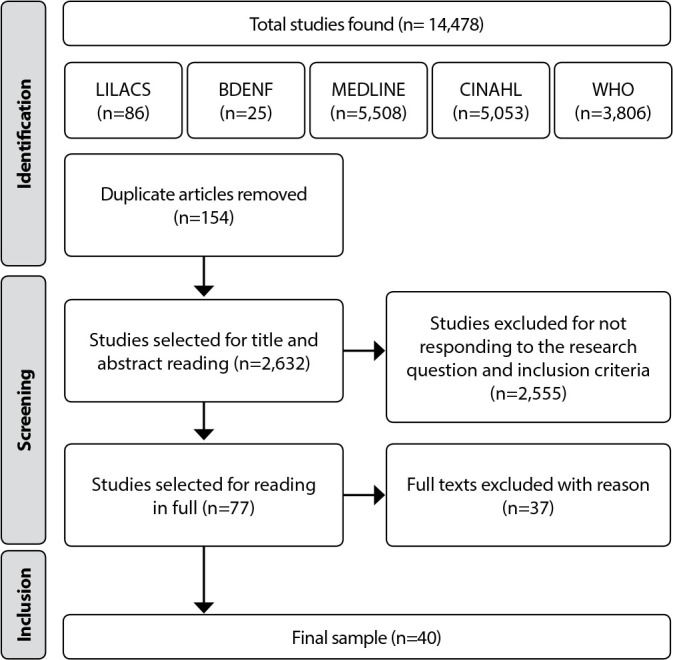
Study selection flowchart, 2022

Initially, 14,478 documents were identified between 2012 and 2022, which were exported to Rayyan in order to systematize sample selection and collection. This software enables the identification of duplicated manuscripts and the formation of inclusion and exclusion categories. These resources were used by two researchers, and disagreements were discussed and reached by consensus. Study selection was determined according to the flowchart shown in Figure 1.

**Figure 2:**
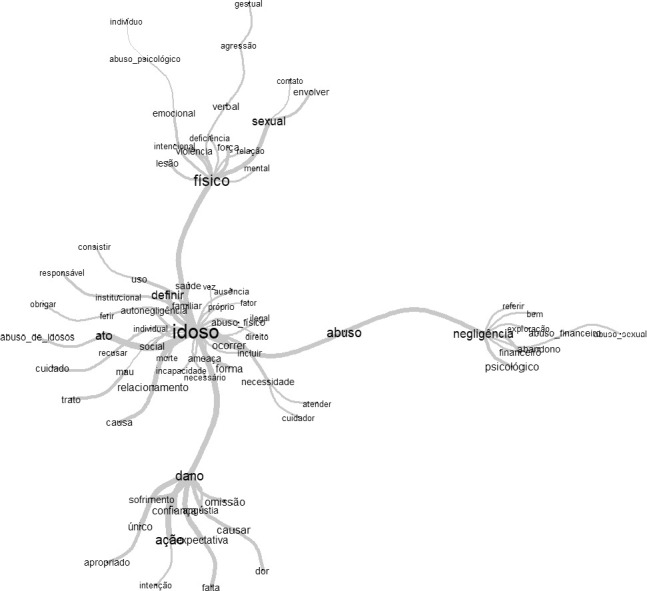
Maximum elder abuse tree representing the empirical references, 2022

#### Data analysis

The integrative review allowed researching, assessing and selecting studies. The manuscripts were read in full and, after careful reading, text fragments were removed, containing expressions or words related to attributes, antecedent and consequent events of EA.

The manuscripts selected to compose the sample were categorized in a spreadsheet with the protocol established for data collection in Microsoft Excel, containing the variables title, year, study design, specific concept of EA, antecedents, attributes and consequents.

The extraction of excerpts from the manuscripts to compose antecedents was done through the question “What events or incidents must occur or exist before the occurrence of the EA phenomenon?”, attributes, “What are the characteristics that express the essence of EA?”, and consequents, “What events or incidents occur as a result of EA?”.

Antecedents and consequents were analyzed in the light of the ecological model, which classifies violence into four risk dimensions: individual; relational; community; and social dimension^(10)^. Attributes were classified according to EA typification.

Only in the last stage, the *Interface de R pour les Analyses Multidimensionnelles de Textes et de Questionnaires* (IRAMUTEQ) was used to determine the empirical references of the analyzed concept. The content from the concept of violence was extracted from the manuscripts, transformed into a textual corpus, analyzed through similarity analysis. As a result, the tool offers a maximum tree that allows the visualization of terms with more emphasis, indicating approximations and distances as well as their respective branches, representing the relationships between terms, by the connectivity line thickness.

During the integrative review execution, it is recommended to determine the level of evidence of the studies included in the sample in order to determine their reliability. Thus, studies with level of evidence LoE I were those from systematic reviews with meta-analysis and studies from randomized clinical trials; LoE II, randomized or experimental trials; LoE III, clinical trials without randomization, experimental studies with non-random selection of subjects; LoE IV, cohort studies/or case-control; LoE V, qualitative systematic review or evidence synthesis reviews; LoE VI descriptive or qualitative studies; LoE VII, opinion studies^(11)^.

## RESULTS

The sample consisted of 40 documents extracted from national and international literature, predominantly classified with level of evidence IV (n=11), descriptive or qualitative studies, according to Chart 1.

**Chart 1:** Classification of articles included in the analysis of the concept of elder abuse, 2022

Author	Title	Year	Study design/ level of evidence
Li; Dong^(12)^	Elder abuse and cognitive function among community-Dwelling older adults: Does abuse history matter?	2022	Cross-sectional V
Ludvigsson et al.^(13)^	Experiences of elder abuse: a qualitative study among victims in Sweden	2022	Cross-sectional V
Dominguez; Storey; Glorney^(14)^	Characterizing Elder Abuse in the UK: A Description of Cases Reported to a National Helpline	2022	Cross-sectional V
Weissberger et al.^(15)^	Elder abuse in the COVID-19 era based on calls to the National Center on Elder Abuse resource line	2022	Cross-sectional V
Souza et al.^(16)^	Factors associated with the risk of violence against older adult women: a cross-sectional study	2021	Analytical IV
Botngård et al.^(17)^	Elder abuse in Norwegian nursing homes: a cross-sectional exploratory study	2021	Analytical IV
Alarcon et al.^(18)^	*Percepção do idoso acerca da violência vivida*	2020	Descriptive VI
Meyer et al.^(19)^	Violence against older women: A systematic review of qualitative literature	2020	Qualitative systematic review V
Hazrati et al.^(20)^	Screening for domestic abuse and its relationship with demographic variables among elderly individuals referred to primary health care centers of Shiraz in 2018	2020	Analytical IV
Saghafi et al.^(21)^	Examining the ethical challenges in managing elder abuse: a systematic review	2019	Systematic review V
Santos et al.^(22)^	*Abuso econômico-financeiro e patrimonial de idosos: um estudo documental*	2019	Cross-sectional V
Yon et al.^(23)^	The prevalence of elder abuse in institutional settings: a systematic review and meta-analysis	2019	Systematic review I
Neuberg et al.^(24)^	Contrasting vantage points between caregivers and residents on the perception of elder abuse and neglect during long-term care	2019	Cross-sectional V
Jeon et al.^(25)^	Gender Differences in the Prevalence and Correlates of Elder Abuse in a Community-Dwelling Older Population in Korea	2019	Cross-sectional V
Maia et al.^(26)^	*A ocorrência da violência em idosos e seus fatores associados*	2019	Cross-sectional V
Santos et ael.^(27)^	Financial-patrimonial elder abuse: an integrative review	2019	Integrative review V
Rodrigues et al.^(28)^	Report of multiple abuse against older adults in three Brazilian cities	2019	Retrospective and longitudinal study IV
Joyce^(29)^	Prevalence and nature of resident-to-resident abuse incidents in Australian residential aged care	2019	Retrospective cohort IV
Mileski et al.^(30)^	Preventing The Abuse Of Residents With Dementia Or Alzheimer’s Disease In The Long-Term Care Setting: A Systematic Review	2019	Systematic review I
Burnes et al^(31)^	Help-Seeking Among Victims of Elder Abuse: From the National Elder Mistreatment Study	2019	Cross-sectional V
Naderi et al.^(32)^	Hospitalized elder abuse in Iran: a qualitative study	2019	Descriptive VI
Castro et al.^(33)^	*Violência contra os idosos brasileiros: uma análise das internações hospitalares*	2018	Analytical IV
Phelan^(34)^	The role of the nurse in detecting elder abuse and neglect: current perspectives	2018	Qualitative systematic review V
Silva et al.^(35)^	*Violência contra idosos: uma análise documental*	2018	Analytical IV
Mahmoudian et al.^(36)^	The design and evaluation of psychometric properties for a questionnaire on elderly abuse by family caregivers among older adults on hemodialysis	2018	Analytical IV
Mawar et al.^(37)^	Association of Physical Problems and Depression with Elder Abuse in an Urban Community of North India	2018	Analytical
Gerino et al.^(38)^	Intimate Partner Violence in the Golden Age: Systematic Review of Risk and Protective Factors	2018	Systematic review I
Oliveira et al.^(39)^	*Violência contra idosos: concepções dos profissionais de enfermagem acerca da detecção e prevenção*	2018	Descriptive VI
Friedman et al^(40)^	Association between Type of Residence and Clinical Signs of Neglect in Older Adults	2018	Analytical IV
Winck et al.^(41)^	*Percepções de enfermeiros da estratégia saúde da família acerca das causas da violência contra a pessoa idosa*	2018	Descriptive V
Cooper et al.^(42)^	Do care homes deliver person-centred care? A cross-sectional survey of staff-reported abusive and positive behaviours towards residents from the MARQUE (Managing Agitation and Raising Quality of Life) English national care home survey	2018	Analytical IV
Eslami et al.^(43)^	Lifetime abuse and perceived social support among the elderly: a study from seven European countries.	2017	Cross-sectional V
Hirt etal.^(44)^	*Representações sociais da violência contra mulheres rurais para um grupo de idosas*	2017	Descriptive VI
McGarry et al^(45)^	Older women, intimate partner violence and mental health: a consideration of the particular issues for health and healthcare practice	2017	Systematic review I
Rodrigues et al.^(46)^	Older adults abuse in three Brazilian cities.	2017	Ecological study V
Pillemer et al.^(47)^	Elder Abuse: Global Situation, Risk Factors, and Prevention Strategies	2016	Scoping review V
Lachs; Pillemer^(48)^	Elder Abuse	2015	Clinical trials without randomization III
Martins et al.^(49)^	Abuse and maltreatment in the elderly	2014	Cross-sectional V
Hernandez-Tejada et al.^(50)^	The national elder mistreatment study: race and ethnicity findings	2013	Cross-sectional V
Yaffe; Tazkarji^(51)^	Understanding elder abuse in family practice.	2012	Review studies V

Chart 2 shows the antecedents related to EA organized according to the ecological model, nine individual characteristics of older adults were found that make them more exposed to violence, eight characteristics of the ecological model’s relational domain, a characteristic classified in the community dimension and six in the social dimension.

**Chart 2:** Antecedents of the analysis of the classified concept according to the ecological model, 2022

Antecedents	
Individual	Advanced age^(17-20,22-25,33-36,43)^, Female^(16-17,19,22,24-26,28-29,33-38,44-46)^; Low education^(17,19,22,33,43-44)^; Single and/or widowed^(17,19,28,43)^; Married^(29,36)^; Limitations/physical dependence^(17,19-20,22-24,26-27,30,34-35,38,40,43-44)^, Psychological and/or cognitive changes^(13,15-17,20-21,23-24,26-27,30-31,36,39)^; Chronic and/or acute health conditions^(19,22,25-26,34-35,38)^; Financially dependent older adults^(19,22,25-26,34-35,38)^; Older adults’ aggressive behavior^(28,31)^.
Relational	Conflicting and/or unstructured family environment^(19,40,42,46)^; Live with family member (son, grandson)^(17,19,28-29,36-37)^; Caused by family member^(24,27-29,34-35,37-38,40,44)^; Male^(18,39)^; Relationship of trust with offender^(34,37)^; Caregiver stress and burnout^(18,22,25,27,30,43)^; Caregiver cognitive and/or psychiatric impairment^(30,34,39)^; Caregiver alcohol and/or drug abuse^(36,38,42)^; Aggression history^(39)^; Intergenerationality^(19,44)^.
Community	Low social support^(16,20,38,44)^; Social isolation ^(16,44)^.
Society	Low income or socioeconomic conditions^(23,25,35,37-38,43-44)^; Gender issues^(29,34-35,44-46)^; Unemployment^(23)^; Difficulty accessing protection services^(16,28,45)^; Negative stereotypes about aging^(35,52)^; Ethnic minority^(38)^; Lack of knowledge of rights^(19)^.

*Note:* Abuse, need, caregiver, assist, older adult, own, absence, abuse, consist, use, responsible, force, act, social, relationship, cause, bad, care, treat, elder abuse, health, turn, define, harm, individual, refuse, institutional, factor, illegal, physical, right, occur, include, death, threat, form, necessary, incapacity, family member, self-neglect, neglect, psychologist, abandonment, sexual abuse, well, financial, refer, exploration, financial abuse, damage, suffering, omission, appropriate, cause, pain, action, expectation, only, intention, lack, trust, anguish, physical, mental, sexual, aggression, psychological abuse, verbal, emotional, injury, contact, involve, deficiency, force, relation, violence, intentional, individual, gestural.

Considering that the occurrence profile of EA is expressed by situations in which the act comes from an individual (offender) on a person (victim), its defining characteristics (attributes) can be expressed in any relation of older adults observed among the last three levels of the ecological model (relationships, community and social). Thus, attributes were not categorized according to the ecological model, as shown in Chart 3.

**Chart 3:** Antecedents of the analysis of the classified concept according to the ecological model, 2022

Attributes	
Psychological violence	Yelling^(18,30,52)^; Swearing and/or insults^(18,22,37,40)^; Nasty and humiliating comments^(18,22,30,36,46,52)^; Threats and/or intimidation^(18,27,30,36,39-40,46)^; Acts of contempt towards older adults and/or their autonomy^(18,27)^; Verbal discussions^(18,27,42,52)^; Rejection of older adults’ religious beliefs^(27)^; Deprivation of rights (freedom) and decisions^(22,27,34,36-37,40)^; Older adults’ isolation from their social life^(19,28,31,36,40)^.
Physical violence	Intentional use of physical force^(20,28,30,35-36,45,52)^ that cause pain or injury^(17)^; Scratches^(20,29)^; Slaps^(39)^; Pushes^(18,37,39,52)^; Burns^(37)^; Pinching^(18)^; Spanking^(37,40,52)^; Kicks^(18,37)^; Punch and/or blow^(39)^; Throwing and/or breaking objects^(18,22,39,52)^; Grasping^(17,27)^; Beating/hurting^(30,37-39)^; Pulling body part^(18,39)^.
Financial violence	Stealing financial resources from older adults^(18,44-45)^; Using resources without their authorization^(18,23,36-38,40)^; Unauthorized use of older adults’ identity for the acquisition of goods or other purposes^(19,22,28,37-38)^; Restricting and/or disregarding older adults’ financial autonomy^(18,25,36-37)^; Destroying older adults’ belongings^(18)^.
Sexual violence	Unwanted touching on or under clothing^(18,37,39)^; Sexual harassment^(18,36-37,39)^; Exhibition of older adults’ body parts^(18,39)^; Digital penetration^(18)^; Unwanted sexual activity^(18,36,40,45)^; Unwanted kiss^(39)^; Unwanted discussion of sexual acts/activity^(18)^.
Neglect	Offering low amounts of insufficient nutrients to older adults^(18,21,30,37,40-41)^; Omission of care for older adults^(18,22,28-29,31,36-37,40,52)^; Low quality of care among institutionalized older adults or ignoring demand from older adults^(22,30-31,37,44)^; Inadequate supply of medications^(18,22,41,52)^; Medication schedule delay^(18,22,41)^.
Abandonment	Not meeting older adults’ affective/emotional needs^(22,37)^; Inattention or lack of personal contact with older adults^(22,37-38)^; Ostracism^(27)^; Older adults feel unwanted^(22,28)^; Government desertion in offering help/protection to older adults^(28,36,40)^; Social insecurity^(28)^; Health care cuts^(27,37)^; Unconcern for older adults’ safety^(22)^.

Chart 4 shows the consequents related to EA organized according to the ecological model. A total of 12 individual consequents were found, three in the relational domain, two related to the community, and four to society.

**Chart 4:** Consequents of classified concept analysis according to the ecological model, 2022

Consequents	
Individual	Damage and/or suffering^(22-24,26,32-38,40,44)^; Psychological disorders^(16,18-19,22,26,29-31,34,36,39,44,46)^; Reversible physical damage^(17-18,23,27,29,31,34,36,39-42)^; Irreversible physical damage^(18,23,34,36,41-42)^; Suicide attempt^(18,33)^; Death^(18,29,36,39,42-43,45-46)^; Loss or decrease in self-esteem and/or self-confidence^(17,19,22,26,34,36)^; Decrease in quality of life^(13,15-16,18,30-31)^; Alcohol abuse^(34)^; Sexually transmitted infection^(34)^; Social introspection^(26,28,31,52)^; Fear^(23,25,28-29,38)^; Unwanted pregnancy^(34)^.
Relational	Expenses related to offender rehabilitation^(18)^; Abortion^(34)^; Aggressor dependence ^(26,28)^; Distancing from family members^(34)^.
Community	Environment of insecurity^(26,28)^.
Society	Damage/loss of human rights or human dignity^(17,22,32-33,35,42)^; Increase in mortality^(17,19,32,41-42,46)^; Medical and hospital costs^(18,32)^; Institutionalizations and/or hospitalizations^(17-18,32,41)^; Social stigma related to marriage^(34)^.

The model case and otherwise were constructed. The model case is understood as a fair example of the concept, demonstrating the concept attributes: MCNP, female, 74 years old, complete elementary school, retired, uses controlled medication. During the nursing consultation, the nurse observes her accentuated thinness, weighing 41 kg with a Body Mass Index of 18.22 and witnesses humiliating and intimidating comments from her husband. The patient mentioned that her husband had a playful manner. On physical examination, the nurse notes burns and bruises in inappropriate places.

Continuing, he observed the edematous genital organ, when touching, complains of pain. Before completion, the husband had to leave the consultation. Voluntarily, the older adult woman mentions that she wants to go to a psychiatrist, but it was not because her husband cut the health plan. When asked about food, the patient reported a low amount of nutrients because her husband uses retirement money for his use, not enough left over. She also mentions that she asks him not to use it because she needs to buy food and medication. The nurse asks her about burns and bruises. At first, she denies it, however, during the conversation she confirms that it was her husband, but asks not to tell him. Tearful, she mentions that she had a bad night last night, where she had sex with her husband and did digital penetration excessively so that she moaned in pain and he would not stop, just when he wanted to.

Otherwise, they are clear examples of “not the concept”. This means that the concepts presented are not attributes, therefore, they do not represent violence against older adults: J.A.M.S, Male, 70 years old, completed higher education, high family income. During the nursing consultation, he asks his wife to be with him, referring to being a person who provides emotional support. The nurse observes harmony between them.

Before completion, the wife had to leave the consultation. The patient mentioned that his wife encourages him to be more and more independent, shows concern for his safety, for his health and with medication schedules. When asked about sexual intercourse, he reported that he has little sexual desire and she responds to his request of not wanting to when asked, pointing out that the relationship is based on respect. It refers that in the relationship there are no aggressions. The nurse asks about finances, and he replies that despite having a joint account, his wife does not interfere with his purchases.

Finally, the empirical references of EA, through similarity analysis, identified were “physical”, “abuse” and “damage”, and it is also possible to observe the term “older adults” in the phenomenon’s central core. In the physical term, through the maximum tree, injury, physical force, intentional act, aggression, gestures and sexual intercourse are observed as operational definition. The term “abuse” is operationally defined as neglect, abandonment, psychological abuse, financial abuse, and sexual abuse. The last empirical reference “damage” has suffering, anguish, pain, omission and breach of expectation as an operational definition.

## DISCUSSION

The widely used definition for EA is that of the World Health Organization (WHO), in which it is characterized as “single or repeated act, or even absence of appropriate action, occurring within a relationship of trust and causing harm, suffering or distress to older adults”^(5)^.

Considering the organization of the concept according to Walk and Avant’s proposition^(8)^ and presentation of similarity, the ramifications corroborate the general definition of EA and provide indicators found for the essential attributes of the phenomenon, antecedents and consequents, which will be further explored in subsequent sections.

### Elder abuse antecedents

The identification of antecedents in the development of a concept analysis provides the researcher with the identification of events or incidents that precede the occurrence of the studied concept and the assumptions implicit in it^(8)^. Allied to this premise, the ecological model was applied in 2011 by US researchers, in which they aimed to relate the risk factors related to EA in institutionalized environments. The authors applied the theoretical model considering its four dimensions (individual, relational, community and social)^(5)^.

Advanced age^(17-19,22-25,33-36,43)^, being female^(16-17,19,22,24-26,29,33-38,44-46)^, having low education^(17,19,27,33,43-44)^, being single and/or widowed^(17,19,28,43)^ or married^(28,35)^ are information collected from sample characterization in research and listed in the literature as risk factors for EA. Considering the ecological model, such characteristics are classified as individual. The world report on violence and health^(10)^ considers that individuals’ biological, personal and historical characteristics reflect on the individual behavior of victims or offenders of violence.

Dependency or limitations of older adults, whether physical^(17,19-20,22-23,28,34-35,37-45)^, psychological^(17,19-20,22-24,26,30-32,34-35,38,40,43-44)^ or financial^(19,22,35,37-38,45)^ as well as chronic and/or acute health conditions^(19,22,25-27,34-35,38)^ are commonly discussed in terms of greater risk of experiencing violent situations.

It is assumed that EA occurs more frequently within the family environment^(29,43)^, and the main offender is one of the members of these older adults’ home^(24,27-29,34-35,37-38,40)^, who, in turn, are also the immediate caregivers of older adults in most situations. Dependent older adults end up generating new demands and responsibilities for their immediate caregivers (usually family members), which can be stressful and generate overload^(18,22,25,30,43)^, and lead to violence.

The model’s second level considers close relationships (marital partners or family members) that raise the risk of violence^(10)^. As previously mentioned, it is in the home environment and close relationships that EA often occurs^(24,27-29,34-35,37-38,40,46)^. Conflicting and/or unstructured family environment is also recognized as a potential risk for EA^(19,40,42,46)^. The essence of a problematic environment emerges from an environment with few limits, separations, lack of commitment between residents, marital conflicts, irresponsibility with activities to maintain a peaceful environment, disrespect and devaluation of older adults^(28,42)^ and drug addiction of children or grandchildren^(36,38,42)^.

Paradoxically, a difficulty encountered in identifying EA is the omission and denial of the violent act by older adults themselves, afraid that the complaint will cause harm to their family member (child, grandchildren or caregiver) in a way that could make their life worse, even if it results in experiencing violence^(40)^.

This discussion ends up culminating in the ecological model’s third level that contemplates the scenario community assessment in search of identifying association with situations of violence^(10)^. The support network for older adults is indicated as a protective factor for older adults in situations of violence^(20,44)^. Within the network, the offer of services in the community is included that provide older adults with security to break the violent cycle and encourage the social inclusion of older adults in social groups that, in addition to improving older adults’ self-esteem, minimizes social isolation which is also a risk factor for EA, are included^(20,44)^.

The model’ fourth level proposes to assess the broad social reasons that determine situations of violence. In this aspect, social inequalities, discrimination, prejudice and cultural norms that reflect violent behavior are discussed^(10)^. In this regard, negative social stereotypes related to aging^(35,52)^, implied by cultural norms that affect older adults’ dignity^(32)^, stigmatize aging as a transversal, static and egalitarian process for all, regardless of context, summed up with stereotypes conveyed by mass media, fostering social ageism growth^(53)^.

Females^(16-17,19,22,24-26,28-29,33-38,44-46)^ are potentially more vulnerable to EA, as listed in the ecological model’s individual dimension. This prevalence affects social contours imbricated in gender distinctions^(29,34-35,44-46)^ observed in the social dimension. The gender distinctions observed among older adults are strongly associated with the cultural and educational aspects in which they developed, making them naturalized^(54)^.

One of the studies^(45)^ included in the sample signals the potentialization of gender distinctions associated with situations of violence by women living in rural areas, in which the social context includes the distinctions of attributions related to sex, in which men are responsible for house income while women are assigned the role of subordinate caregiver. Furthermore, the rural environment makes it more difficult to identify EA cases and access services and the victim protection network^(45)^.

### Elder abuse attributes

The attributes of a concept analysis consist of characteristics that define the occurrence of the studied phenomenon, being useful to carry out the differential diagnosis in the medical sciences. They also provide support to identify which are the differential attributes of the outcome studied as well as nonspecific attributes, but which are related to the concept. Walker and Avat^(8)^ also indicate that the volume of information provided by the analysis can be large, making it necessary to make decisions about which characteristics are essential for understanding the concept.

The attributes presented in this review do not exhaust the literature related to EA, but provide an understanding of strong characteristics that support the identification of its occurrence. Thus, the attributes were categorized according to the type of violence (psychological violence, physical violence, sexual violence, financial and economic violence, neglect, self-neglect and abandonment) and the level of the ecological model in which the attribute was classified.

Psychological violence perpetrated against older adults consists of verbal or gestural attacks with the purpose of limiting social interaction, isolating, humiliating or causing fear in older adults^(19)^. Hazrati et al.^(20)^ add that this definition is the result of inadequate responses to feelings and emotions.

Psychological violence is the most prevalent type of EA^(28)^. However, its identification is challenging since its occurrence happens in a domestic environment. Older adults are commonly afraid to indicate being victimized by violent acts^(40)^ and the social normalization of psychologically violent experiences, such as name-calling and derogatory words in everyday life.

Psychological violence precedes more severe acts of violence, thus indicating the need to pay more attention to signs of emotional abuse^(4)^. Among the relationships of older adults in all instances of ecological modeling, signs of abuse can be observed such as yelling^(18,30,52)^, swearing and/or insults^(18,22,37,40)^, nasty and humiliating comments^(18,27-28,30,36,40,52)^, threats and/or intimidation^(18,27,30,36-37,39-40,46)^, acts of contempt towards older adults and/or their autonomy^(18,22)^, verbal discussions^(18,27,30,42,52)^, rejection of older adults’ religious beliefs^(22)^, deprivation of rights (freedom) and decisions^(22,27,34,36,38,40)^ and older adults’ isolation from their social life^(19,28,31,36,40)^.

Physical violence, in turn, consists of “the use of physical force to injure, cause pain, disability or death or to compel older adults to do what they do not want to do”^(54)^. It is characterized by physical injuries capable of causing pain or injury^(17,20,32-33,39)^, scratches^(20,29)^, slaps^(39)^, pushes^(18,37,39,52)^, burns^(37)^, pinching^(18)^, spanking^(37,40,52)^, kicks^(18,37)^, punches and/or blows^(39)^, throwing and/or breaking objects^(18,27,39,52)^, grasping older adults^(17,29)^, beating/hurting^(30,37-38,40)^ and pulling body parts from older adults against their will^(18,39)^.

Financial violence commonly occurs concomitantly with other forms of violence, and its occurrence is widespread and known in the Brazilian context, although it is believed that there is a lot of underreporting of cases^(27)^. A study carried out at the Police Station for Security and Protection of Older adults, northeastern Brazil, in Teresina, located in Piauí, identified a growing trend in the recording of financial violence compared to other types of abuse against older adults^(22)^.

Predominantly affects older adults’ homes through fraudsters in banks, health plans and stores^(27)^. The main attributes that indicate the occurrence of financial violence are clear expressions of theft of financial resources from older adults^(18,44-45)^, use of resources without their authorization^(18,23,36-38,40)^, unauthorized use of older adults’ identity for the acquisition of goods or other purposes^(18,27-28,36-38)^, restricting and/or disregarding older adults’ financial autonomy^(25,27,36-37)^ and destroying older adults’ belongings^(18)^.

Ageism related to older adults’ sexuality and sexual activity is a reflection of a cultural tendency that assumes the age group’s asexuality. The extrapolation of this prejudice does not perceive older adults as likely victims of sexual violence. Since they do not practice consensual sex, it is assumed that they are not the target of sexual acts without consent. A study developed in the United Kingdom found that most victims were aged between 60 and 69 years; offenders were younger than the victim (50 to 59 years old); offenders were known people; and the most common place of occurrence was the home and nursing homes^(55)^.

Sexual abuse is conceptualized as “sexual acts or games of a homo or heterorelational character that use older adults in order to obtain excitement, sexual intercourse or erotic practices through grooming, physical violence or threats”^(54)^. It is characterized by unwanted touching on or under clothing^(18,37,39)^, sexual harassment^(18,36-37,39)^, exhibition of older adults’ body parts^(18,39)^, digital penetration^(18)^, unwanted sexual activity^(18,36,40,45)^, unwanted kiss^(39)^, and unwanted dialogue about sexual acts/activity^(18)^.

The discussion of older adult negligence and abandonment are commonly explored in an associated way; however, it is necessary to clarify the difference between both phenomena. While negligence consists in the omission of essential care for the maintenance of older adults’ health by those responsible (formal or informal caregiver), abandonment, in turn, consists in abandonment of older adults by those responsible (family, institution or government) in providing assistance to older adults in need of protection^(54)^.

Negligence occurs very frequently during older adults’ institutionalization^(22,30-31,38,41)^. It may also occur at home^(20)^, during the care offered by family members^(18,20,23)^ or by a formal caregiver^(19,23)^. It is often typified as intentionally supplying insufficient nutrients for older adults’ needs^(18,22,37)^, omission of care for older adults^(18,22,27-28,30,36-37,40-41)^, low quality of care among institutionalized older adults or ignoring demand from older adults^(26,30-31,37,41)^, greater supply of medications than necessary for older adults’ treatment^(18,22,41,52)^ or delay in their administration time^(18,22,41)^.

Older adults are supported by several legal norms that guarantee them social rights and duties; however, the invisibility that affects this specific group ends up generating older adult social exclusion and abandonment. Abandonment will be configured as the omission of others to meet their legal responsibilities of assisting older adults^(54)^, including their affective dimensions.

In the typification of abandonment, the essential attributes are actions that do not meet older adults’ affective/emotional needs^(27,37)^, inattention or lack of personal contact with older adults^(22,37-38)^, ostracism^(37)^, older adults feel unwanted^(22,38)^, government desertion in offering help/protection to older adults^(28,36,40)^, social insecurity^(28)^, health care cuts^(22,37)^ and unconcern for older adults’ safety^(22)^.

### Elder abuse consequents

The definition of a phenomenon’s consequents consists of identifying the incidents resulting from the occurrence of the concept, its relationship with events that are commonly marginalized and generating new evidence^(8)^. The studied concept of EA involves consequents within the ecological model’s four levels^(5)^.

Among the individual consequents observed in the manuscripts, it was possible to observe a significant amount of evidence that points to damage and/or suffering^(23-24,33-37)^ in older adults victimized by violence. This is associated with the definition proposed by the WHO for EA, which includes as a result any act that results in harm or suffering^(5)^.

When immersing in search of a better understanding of the damage and suffering that EA causes, it is possible to verify that psychological consequences appear in the form of psychological disorders^(16,18-19,22,28-31,34,36,39,42,44)^, loss or decrease in self-esteem and/or self-confidence^(19,27,36,44)^, decrease in quality of life^(17,19,22,25,34,36)^, social introspection^(26,28,31,52)^, fear^(23,25,28-29,38)^, suicide attempt^(18,33)^ and even death^(18,29,36,39-40,42-43,45-46)^.

The emergence of depression in older adults who experience situations of violence^(16,56-57)^ is a consequence of great impact on affected older adults’ health. Research developed in São Paulo^(57)^ describes in its results that older adults who indicated vulnerability to exposure to violence had a mild to severe depression picture, adding to perceived stress.

Early diagnosis and adequate treatment of symptoms related to depression in older adults include understanding by the health team the main risk factors to which these older adults are exposed, including sociodemographic and health conditions^(57)^, more severe than depression can cause, such as suicidal ideation and even suicide^(56)^.

Limitations and physical disability, on the one hand, consists of a risk factor^(16-17,19-20,22-23,28,30-32,34-35,37-45)^ for EA, and, on the other hand, older adults without limitations or physical damage may present them due to violent acts, whether reversible^(17-18,23,27,29,31,34,36,39-42)^ such as injuries^(17,19,41)^, injuries^(29)^ and pressure injuries^(40)^, and/or irreversible^(18,34,36,41-42)^, such as functional disability^(12,15)^ and HIV^(19)^.

The assessment of older adults’ functional capacity is commonly determined by assessing whether or not older adults are dependent on carrying out basic (eating, sphincter control, transfers, ability to dress, take a shower and use the toilet) and advanced (preparing meals, doing housework, handling money, using the telephone, taking medications, shopping and using transportation) activities of daily living. To this end, two widely disseminated instruments are used: the Katz Index, for basic life activities, and the Lawton Scale, for intermediate life activities.

However, the proximal relationships of older adults also have consequences resulting from EA, which represents the ecological model’s second level^(10)^. In the review, high expenses were identified with short-term rehabilitation of offenders^(17)^, situations of breaking the mother-child binomial through abortion^(19)^, dependency on older adults of EA offenders^(16,22)^ and distancing from family members^(19)^.

Insecurity^(22,29)^ was unveiled as a community consequence of EA occurrence, which encompasses relationships with the community in which the phenomenon of violence occurs. This feeling of insecurity can happen in the team that assists older adults both in denouncing and reporting the case^(18)^.

This feeling of social insecurity and fear of offenders by the team ends up generating underreporting of EA cases. Some professionals are able to identify the situation of violence, but prefer that it be reported by older adults or a family member^(58)^, enhancing the silencing of the phenomenon.

The work carried out by health teams takes place in fixed territories, and the team’s work process consists of forming a bond with the community. This relationship between the professional and the community ends up generating fear and insecurity for professionals to actively search, report and denounce cases of EA, since aggressors can blame them and leave them in a situation of risk and social vulnerability^(58)^.

EA also incurs consequences that impact society as a whole, this being the ecological model’s fourth structural level^(10)^. Within this perspective, it was possible to identify social impacts arising from the phenomenon as increase in mortality^(17,19,32,41-42,46)^, damage to dignity/human rights^(17,27,32-33,36,42)^, increase in medical and hospital costs^(18,32)^, institutionalizations and/or hospitalizations^(17-18,32,41)^ and increase of social stigma related to marriage^(34)^.

A Brazilian study proposed to assess the costs of hospitalizations due to situations of abuse in people aged 60 years or over reported between 2010 and 2019, in which the results indicate that BRL 99,451.27 was made available for this purpose. Of these, 83.93% (BRL 83,472.17) were allocated to hospital services and 16.07% (BRL 15,979.10) to human resources^(59)^. These data corroborate the argument of the social impact of EA.

Aging in Brazil involves multiple consequences for older adults, as social disparities and stigmas associated with aging become more apparent, such as the devaluation of human dignity enhanced by capitalism, in which the social value of individuals is essentially linked to their production capacity, making older adults a social burden at the time of their retirement.

From this point of view, the consequences arising from violent acts perpetrated against older adults imply social inequalities that walk on older adults’ evaluative perspective and their observed productivity in capitalism. On the other hand, the dismantling of aging as a process of physiological changes and transitions will demand adaptations from the social system in order to provide them with dignity and quality during aging.

### Study limitations

A study limitation may include the failure to carry out the model case construction stage and additional cases, which may be the proposal for a new study.

### Contributions to nursing, health, and public policies

This analysis provides scientific support for understanding and discussing EA as a relevant phenomenon, also enabling theoretical advancement in health. The clarification of the concept provides knowledge of relevant empirical data for constructing instruments, protocols, lines of care for older adults victims of violence, public policies, assistance programs for health promotion and EA prevention.

## FINAL CONSIDERATIONS

The present study enabled the analysis of the concept of violence, allowing greater refinement of the multidimensionality of the phenomenon under study, since it can present itself in different typifications and from each of them to present multiple characteristics.

Concept analysis data provide theoretical and scientific content for combating EA, revealing terms that characterize the antecedents, attributes and consequents, in addition to empirical references with operational definitions, providing understanding and deepening of the theme.

It is suggested to carry out other analyzes of the specific concept for each type of EA, in order to further refine the discussion of the findings of this study and then make it easier for health professionals to better understand its occurrence, its risk factors, its defining characteristics and the consequents arising from injury occurrence.
